# Clinical case review: A method to improve identification of true clinical and radiographic pneumonia in children meeting the World Health Organization definition for pneumonia

**DOI:** 10.1186/1471-2334-8-95

**Published:** 2008-07-21

**Authors:** Taneli Puumalainen, Beatriz Quiambao, Erma Abucejo-Ladesma, Socorro Lupisan, Tarja Heiskanen-Kosma, Petri Ruutu, Marilla G Lucero, Hanna Nohynek, Eric AF Simoes, Ian Riley, the ARIVAC Research Consortium

**Affiliations:** 1 National Public Health Institute, Mannerheimintie 166, FIN-00300 Helsinki, Finland; 2 Research Institute for Tropical Medicine, Metro Manila, The Philippines; 3 University of Colorado, Denver, USA; 4 University of Queensland, Brisbane, Australia

## Abstract

**Background:**

The World Health Organization's (WHO) case definition for childhood pneumonia, composed of simple clinical signs of cough, difficult breathing and fast breathing, is widely used in resource poor settings to guide management of acute respiratory infections. The definition is also commonly used as an entry criteria or endpoint in different intervention and disease burden studies.

**Methods:**

A group of paediatricians conducted a retrospective review of clinical and laboratory data including C-reactive protein concentration and chest radiograph findings among Filipino children hospitalised in the Bohol Regional Hospital who were enrolled in a pneumococcal vaccine efficacy study and had an episode of respiratory disease fulfilling the WHO case definition for clinical pneumonia. Our aim was to evaluate which disease entities the WHO definition actually captures and what is the probable aetiology of respiratory infections among these episodes diagnosed in this population.

**Results:**

Among the 12,194 children enrolled to the vaccine study we recorded 1,195 disease episodes leading to hospitalisation which fulfilled the WHO criteria for pneumonia. In total, 34% of these episodes showed radiographic evidence of pneumonia and 11% were classified as definitive or probable bacterial pneumonia. Over 95% of episodes of WHO-defined severe pneumonia (with chest indrawing) had an acute lower respiratory infection as final diagnosis whereas 34% of those with non-severe clinical pneumonia had gastroenteritis or other non-respiratory infection as main cause of hospitalisation.

**Conclusion:**

The WHO definition for severe pneumonia shows high specificity for acute lower respiratory infection and provides a tool to compare the total burden of lower respiratory infections in different settings.

**Trial registration:**

ISRCTN62323832

## Background

The World Health Organization (WHO) has developed standard case management guidelines to reduce the two million deaths, or 20 percent of all child deaths, caused by pneumonia through early diagnosis and treatment [1]. A meta-analysis showed that these guidelines, if effectively implemented, result in significant reduction in mortality: 24% (95% CI 14–33%) in total mortality and 36% (95% CI 20–48%) in pneumonia-related mortality in children aged 0 to 4 years in developing countries with infant mortality over 90 per 1,000 live births [2]. Many countries have now adapted these guidelines as part of national acute respiratory infections control and Integrated Management of Childhood Illnesses (IMCI) programs.

The WHO guidelines define pneumonia as an acute disease episode with cough or difficult breathing combined with fast breathing with age specific cut-off values for increased respiratory rate. Children with lower chest wall indrawing classified as severe pneumonia are referred for evaluation and possible in-patient care. While it is estimated that these criteria detect over 80 percent of children that require antibiotic treatment for probable bacterial pneumonia or hospital care for severe disease, 20 to 30 percent of children fulfilling the criteria receive unnecessary antimicrobials for non-severe viral respiratory infection [3]. This is especially true for children with expiratory wheezing due to asthma, bronchiolitis or other viral respiratory infections who are often misclassified as pneumonia requiring antimicrobial treatment and referred for in-patient care [4].

Another problem of the low specificity of the WHO definition for pneumonia is its use as study enrolment criteria or one of the study endpoints in disease burden and intervention studies. For example, studies evaluating the efficacy of new conjugated vaccines against *Streptococcus pneumoniae *show these vaccines are effective in reducing invasive pneumococcal disease and radiographic pneumonia, but the efficacy is low for the WHO-defined clinical pneumonia [5,6].

We carried out a randomised, double-blinded, placebo-controlled study to evaluate the efficacy of an investigational 11-valent pneumococcal conjugate vaccine against childhood pneumonia in the Philippines. We used the WHO definition for pneumonia as entry criteria for clinical data collection from study subjects hospitalised and as a secondary endpoint for vaccine efficacy analysis. This report describes, without un-blinding the study randomisation code, results of a retrospective review of clinical, laboratory and radiological data from study subjects hospitalised with an illness that on admission fulfilled the WHO definition for pneumonia. Our aim was to describe the clinical methods used in our vaccine study and evaluate the use of WHO definition for clinical pneumonia in a South East Asian paediatric hospital setting. We attempted to improve the specificity of diagnosis of WHO defined clinical pneumonia by classifying the disease episodes into International Classification of Diseases, version 10 (ICD-10)-classification system categories for pneumonia which then could be used to analyse the vaccine efficacy. The results are discussed in light of possible different diagnostic approaches for respiratory infections, which could be utilised in pneumococcal or other vaccine and disease burden studies.

## Methods

### Study setting

The study was conducted in six municipalities in Bohol Province in central Philippines. At the beginning of the vaccine study in 2000, the predominantly agricultural area covering 357 Km^2 ^had a total population of 149,000. There is no malaria and practically no HIV in Bohol [7]. In 2005, the Gross National Income of the Philippines was 1,300 USD/capita. The infant mortality rate (IMR) in Bohol during years 1999–2002 was 28/1,000 live births, with the major causes of child deaths being pneumonia and diarrhoea [7].

### Study subjects

This study to evaluate the efficacy of an 11-valent mixed carrier tetanus protein or diphtheria toxoid conjugated pneumococcal vaccine against radiographic pneumonia in children aged 6 weeks to 23 months started in July 2000. All children born in the study area and healthy enough to receive their first diphtheria, tetanus, whole-cell pertussis vaccine (DTwP) dose according to the national Expanded Program on Immunization (EPI) program were offered study participation. The vaccines administered included Bacillus-Calmette-Guerin (BCG) at 2 weeks of age, 3 doses of DTwP and *Haemophilus influenzae *type b (DTwP//PRP-T) conjugate vaccine, plasma derived hepatitis B vaccine and study vaccine or placebo (normal saline) starting at age of 6 weeks to < 6 months with a minimum interval of 4 weeks between the doses. Measles vaccine was given at 9 months of age.

### Case ascertainment for pneumonia

Patients described in this paper were evaluated at the Bohol Regional Hospital, which is a 250-bed tertiary government hospital managing approximately 85% of children with severe infections aged less than 5 years from the study area [8]. Most of the children were walk-in patients without referral from the primary health care clinics. All children were seen within 30 minutes of arrival by a study nurse who screened whether the patient's age (6 weeks to 23 months), residency (six study municipalities) and symptoms met the criteria for data collection. The eligibility was then immediately reconfirmed by a physician. A community acquired pneumonia was defined as pneumonia with onset prior to or less than 72 hours following admission to hospital. The WHO criteria for non-severe pneumonia included: a history of cough and/or difficult breathing of less than 3 weeks duration, with (a) increased respiratory rate (Rate ≥ 60/min if age <2 months, ≥ 50/min if age 2–11 months and ≥ 40/min if age 12–59 months); (b) lower chest wall indrawing (severe pneumonia); or (c) cyanosis and/or inability to feed or drink (very severe pneumonia). In case of expiratory wheezing on auscultation the child was first given three doses of an inhaled bronchodilator, and the eligibility was re-assessed 30 minutes after the last dose. The child was further evaluated according to the study procedures if the signs and symptoms of WHO-defined pneumonia were still present after the bronchodilator treatment. New admission due to a disease meeting the WHO pneumonia criteria within 14 days of the previous evaluation was considered to belong to the same disease episode. The haemoglobin oxygen saturation at room air was measured with BCI 3303 oximeter (Waukesha, USA) after stabilization of the reading. A blood sample was drawn for bacterial culture, white blood cell count (Advia 60 automatic analyzer, Bayer, USA), and serum. The bacterial cultures were conducted by using standard methods [Methods described in detail in references [9] and [10]]. Regular quality control of bacterial culture techniques and findings were conducted by the Research Institute for Tropical Medicine in the Philippines and National Public Health Institute in Finland. Coagulase negative staphylococci, Bacillus, diphtheroids and gram negative non-fermenting organisms were considered contaminants. The serum samples were transported frozen to Finland for analysis of C-reactive protein by immunoturbidometry and radioimmunoassay (Hitachi Modular P1-analyser, Hitachi Ltd, Tokyo, Japan) at the Helsinki University Central Hospital. All patients were subjected to chest radiograph which was interpreted by using a reading process developed by the WHO [11]. The radiographs were also interpreted separately using a structured questionnaire by an independent pediatric radiologist blinded to the clinical data. Survival of all study subjects was checked by the study team at the age of 23 months, or following the termination of the study data collection in December 2004.

### Quality assurance of case ascertainment

The study nurses and physicians were trained in the WHO pneumonia-management algorithm [12,13] and were monitored weekly for accuracy and consistency of measurement of respiratory rate, chest indrawing and other signs of pneumonia. The intensive monitoring kept the inter-observer variation in pneumonia severity assessment at less than 10% during the study period. All data were double entered into the study database. An independent monitoring team consisting of clinical research associates constantly checked the integrity of all clinical study data.

### Review of patient data by the clinical review team

The retrospective review of clinical data collected during 2000 to 2004 from each disease episode was carried out from November 2003 to March 2005 by a team consisting paediatricians familiar with the study setting (TP, BQ, EA-L, SL, TH-K). Structured data from the study database (as shown in Table 1) and non-structured admission and daily follow-up data from the hospital records, as well as results of laboratory and radiology investigations were used as source data. The review team utilised the International Classification of Diseases, version 10 (ICD-10) coding system to record final diagnoses for each disease episode. Disease episodes with clinical signs and symptoms of infection and infiltrates or pleural fluid in chest radiograph were classified as pneumonia whereas those episodes with normal or missing chest radiograph as clinical lower respiratory tract infection. The review team also further classified the pneumonia episodes according to the probable aetiology, i.e. to bacterial, mixed bacterial-viral and viral infections, by using the best clinical judgement based on clinical data, C-reactive protein concentration, white blood cell count, radiographic appearance and response to treatment with or without antimicrobials. Underlying medical conditions such as malnutrition and congenital abnormalities were also registered. The most important medical condition leading to hospitalisation was regarded as the primary diagnosis. The intra-observer variability of the review process was evaluated in approximately 10 percent of episodes by repeating the review several months later blinded to the results of the first review.

### Ethical review and approvals

The parents or guardians of study subjects signed an informed consent before enrolment to the vaccine study. All therapeutic decisions were done independently by regular hospital physicians not belonging to the study team. The study was conducted in accordance with the latest South African revision of the Declaration of Helsinki, ICH Good Clinical Practice, and local regulatory requirements.

The concept of the trial submitted under title "Phase III Trial on the Efficacy of an 11-Valent Pneumococcal Conjugate Vaccine in the Reduction of Severe and Very Severe Pneumonia Among Filipino children Under 2 Years of Age" was approved by the Ethical and Institutional Review Board of the Research Institute of Tropical Medicine, the Philippines in November 1998. The final version of the trial protocol was approved in June 2000.

### Statistical analysis

This report describes the clinical picture of pneumonia requiring hospitalisation and the method of clinical review used in our vaccine efficacy study context. No statistical hypothesis testing was conducted. We calculated Kappa coefficient for the reproducibility of the review.

## Results

The planned cohort of 12,194 infants was enrolled the vaccine efficacy study by December 2003 (78% of the total birth cohort, 92% of the non-transient population eligible for the study). Overall, 98.7% of the enrolled subjects received 3 doses of study vaccine. The median age at vaccination was 1.8, 2.9 and 3.9 months for first, second and third dose, respectively. Vaccine and placebo groups did not differ in key baseline socio-economic indicators (data not shown). The data on all hospital admissions and other important medical events were collected until study termination visit at 23 months of age (N = 8,780) or end of study follow up at the end of December 2004 (N = 2,366). Altogether 64 children died and 984 children were lost to follow up due to migration or were withdrawn from the study for various reasons.

This analysis covers 1,195 disease episodes fulfilling the WHO criteria for pneumonia among 821 children (60% boys) who were hospitalised at the Bohol Regional Hospital. The age distribution is shown in Table 1 [see Additional file 1]. According to the WHO pneumonia severity classification algorithm, 290 episodes (24%) were non-severe pneumonia i.e. without chest indrawing or other danger signs, 785 (66%) were severe pneumonia presenting with chest indrawing and 120 (10%) were very severe pneumonia with either inability to drink or central cyanosis. A blood culture was obtained in 90% of episodes. We detected 13 (1.1% of episodes) invasive bacterial infections. The most common bacterial pathogens included *Staphylococcus aureus, Streptococcus pneumoniae *and *Salmonella *Typhi. Chest radiographs from 182 (15.2%) episodes showed abnormal findings which met the WHO criteria for probable bacterial pneumonia (Primary endpoint consolidation). The mean duration of hospitalisation was 3.2 days (Range 0 to 32 days). Altogether 19 patients died while in hospital or within 14 days from discharge (Case fatality rate 1.6%).

The distribution of key clinical, laboratory and radiology findings in each of the WHO pneumonia severity categories is shown in Table 1 [see Additional file 1]. In episodes of severe, but not very severe pneumonia, 51% of children presented with expiratory wheeze compared to 9.3% in the non-severe and 40.8% in the very severe pneumonia categories. Very severe pneumonia was associated with highest probability for abnormal radiograph and hypoxemia, as well as the highest case fatality rate: 9.2% compared to 0.3% in non-severe pneumonia and 0.5% in severe pneumonia categories.

The WHO classifications of severe and very severe pneumonia identified acute lower respiratory infection very well (95% and 85% respectively) whereas the non-severe pneumonia classification identified only 49% of subjects with ALRI. According to the retrospective review of clinical, laboratory and radiographic data, 402 episodes (33.6%) had radiographically confirmed pneumonia as the major cause of hospitalisation (Table 2) [see Additional file 1]. Bacteriologically confirmed bacterial pneumonia or probable bacterial pneumonia was the diagnosis in 8.6%, 11.5% and 15.9% of episodes in non-severe, severe and very severe WHO pneumonia categories, respectively. Viral lower respiratory infection i.e. viral pneumonia or bronchiolitis was most common in the severe pneumonia category: 37.5% compared to 14.5% and 25.9% in non-severe and very severe pneumonia categories, respectively.

A total of 203 episodes (17.0%) were evaluated as not having a lower respiratory infection as the main reason for hospitalisation. Non-respiratory diseases, of which acute gastroenteritis was the most common, accounted for 12.7% of hospitalisations. The other non-respiratory diagnoses included a broad range of paediatric diseases such as acute febrile infections with seizures, skin infections, urinary tract infections and 9 cases of central nervous system infections.

Episodes which fulfilled the WHO criteria for non-severe pneumonia had the highest likelihood for having a non-respiratory disease as the main diagnosis. However, in 15 of the 37 episodes of acute gasteroenteritis the child had also signs of lower respiratory infection (3 with radiographic abnormalities). In total, seven of the 290 (2.4%) of episodes were diagnosed as sepsis or meningitis. In the severe pneumonia group, other than lower respiratory infection diagnoses were rare. The 12 episodes of gasteroenteritis included one episode, in which the patient also had a diagnosis of viral pneumonia. The 11 other episodes presented with chest indrawing on admission, but this could have been due to dehydration or simultaneous lower respiratory infection which did not show any radiographic abnormalities. Only in two episodes, the final diagnosis was meningitis or sepsis without pneumonia. The other diagnoses in the very severe pneumonia group included five episodes of sepsis, four episodes of central nervous system infections and three episodes of acute gastroenteritis. One episode was classified as an upper respiratory infection.

In order to ensure the reproducibility of the review, 111 episodes (9.3%) were re-evaluated several months later by the same review team who were blinded to the results of the first review round. The team assigned the same ICD-10 coded diagnosis in 79 of the 111 episodes evaluated (71.2%). Additional 21 episodes (18%) had diagnosis in the same disease group, but the ICD-10 coding differed (e.g. J22 non-specified lower respiratory infection vs. J21.9 bronchiolitis). A total of 37 episodes were assigned a pneumonia group diagnosis in the first review compared to 44 in the second review (Kappa coefficient 0.79). According to both reviews 93 episodes had a diagnosis belonging to any of lower respiratory tract infection categories (Kappa 0.87).

## Discussion

Our study demonstrates that in this population of Filipino children the WHO definition of clinical pneumonia captures a broad spectrum of respiratory infections varying from mild upper respiratory infections to severe bacterial pneumonia. In total, one third of episodes showed radiographic evidence of pneumonia and one third of these (11.2% of the total) were classified by the group of paediatricians as definitive or probable bacterial pneumonia. The severity of the disease correlated well with the WHO pneumonia severity algorithm. The non-severe pneumonia group had a higher probability for upper respiratory and non-respiratory group diagnoses than the severe or very severe pneumonia groups which identified very well the lower respiratory and other severe infections.

The findings of this study are in conformity with other studies conducted in the Philippines [14,15] and in other Asian countries [4] reporting a similar clinical picture in patients with a WHO-defined pneumonia. The case fatality rate of 0.5% observed in the severe pneumonia group was similar to that in a recent multi-centre study evaluating antimicrobial treatment alternatives for WHO-defined severe pneumonia [16], but lower than the 2.1% and 12% mortality described in Bohol, the Philippines during 1990's and in Lombok, Indonesia among children hospitalised for acute lower respiratory infection, respectively [17,18]. This may reflect the different patterns in care seeking behaviour and access to care.

The microbial aetiology of respiratory infection is difficult to determine, as the sensitivity of tests, especially bacterial cultures, is low, and mixed bacterial and viral infections are common. The chest radiograph and non-specific laboratory markers such as C-reactive protein, procalcitonin and white blood cell count provide some guidance, but the results overlap significantly in viral and bacterial pneumonia [19,20]. In this study the WHO classification of non-severe, severe and very severe pneumonia appeared to correlate with disease severity, but poorly with the probability of definitive or probable bacterial aetiology for infection. The distributions of C-reactive protein concentration and blood leukocyte count did not suggest major differences in proportions of episodes with bacterial aetiology in the different severity categories of pneumonia. Despite special attention to laboratory techniques the ratio of positive blood culture findings was low in all pneumonia severity categories. This may be partly explained by Hib vaccination provided to all study participants and pre-hospital antimicrobial treatment, which was, according to a parental questionnaire, 35% in our previous epidemiologic study conducted in Bohol during 1995–99 [9]. Nevertheless the overall ratio of invasive pneumococcal infections is far lower than those seen in studies in Africa [21]. In this study 15.2% of episodes showed a primary endpoint consolidation in chest radiograph. The frequency of chest radiograph findings increased with the pneumonia severity. The Gambian pneumococcal study, which used similar WHO criteria for case ascertainment, reported a primary endpoint radiographic finding in 18.1% of hospitalized children [5]. However, in the Gambian study, 4.2% of hospitalized patients had a blood culture or CSF positive for *Streptococcus pneumonia *compared to 0.3% in this study population.

Other studies have reported 65% to 90% specificity of the WHO pneumonia criteria for lower respiratory infection [3,12,13,21]. In our study, the retrospective clinical review demonstrated that in 17% of episodes the child showed signs and symptoms of WHO-defined pneumonia, but the main condition prompting the decision to admit the patient for hospital care was not the acute lower respiratory infection. Largely this consists of patients with non-severe pneumonia, of which over half had another concomitant disease that was the main reason for hospitalisation. According to the WHO guidelines the children with non-severe pneumonia should not be referred for hospital care, but provided with oral antibiotics for acute respiratory infection. Among children with severe pneumonia presenting with chest indrawing, the frequency of concomitant disease prompting the decision for admission was low (< 5%). The episodes of very severe pneumonia included, in addition to lower respiratory infections, also other severe infections. This finding supports the current practise of including these patients to the IMCI guidelines category of severe disease needing urgent referral to hospital care.

We noted expiratory wheeze in half of episodes of WHO defined severe pneumonia. This finding emphasises the importance of including the management of wheeze to the current pneumonia management guidelines. It is not known whether all children with wheeze, presumable caused by a viral infection, require antimicrobial therapy and, if not, which additional clinical findings would support withholding antibiotics [3,4]. The WHO currently recommends that children without previous history of wheeze who develop a lower respiratory infection with wheeze and tachypnoea may have a bacterial or mixed bacterial-viral infection, and should always be treated with antibiotics for suspected bacterial pneumonia [23]. The findings from the South African pneumococcal study, which reported 31% efficacy of the vaccine against pneumonia associated to respiratory viruses support this strategy [24]. The situation may, however, be different in many Asian countries where the incidences of wheezing diseases and paediatric asthma have increased and mortality attributable to severe respiratory infections has decreased [3,25,26].

A limitation of this study is the uncertainty of the role of the pneumococcal conjugate vaccine in modifying the clinical picture of respiratory infections. Other pneumococcal vaccine efficacy studies suggest that the vaccine may prevent 20–36% of radiographic pneumonia [5,6]. It is thus possible that viral respiratory infections are enriched in this patient population. We assumed that in 5% of episodes where a chest radiograph was not obtained the clinical review diagnosis was not pneumonia. While probably missed some infiltrates we do not believe that the overall interpretation of the data would have changed significantly. The diagnostic procedures conducted in this study represent practices available in many hospitals in South East Asian countries. The results of this study are not, however, directly applicable to other health care settings, in which the clinical picture of WHO-defined clinical pneumonia may be different. The reproducibility of our clinical case review process was high in our own study settings, but it is difficult to know if this process, based primarily on clinicians' best judgement, could be repeated in similar manner elsewhere. Whether the classification according to probable aetiology of infection was successful can be evaluated once we analyse the vaccine efficacy in different ICD-10 categories of respiratory infections and complete the on-going testing of respiratory viruses from nasopharyngeal samples collected from hospitalized study children.

The WHO case management algorithm for pneumonia provides an important tool for managing respiratory infection in resource poor settings. It also provides a tool to compare the total burden of respiratory infections in different communities, but should not, without additional microbiological evidence, be used to compare the burden attributable to specific respiratory pathogens. This study suggests that the category of WHO-defined severe pneumonia is very specific for lower respiratory infections whereas the non-severe pneumonia category includes, at least in hospital settings, a large proportion of other diseases.

## Conclusion

The WHO definition for clinical pneumonia captures a broad spectrum of different paediatric respiratory diseases. The WHO classification of non-severe, severe and very severe pneumonia correlated well with disease severity, but poorly with the probability of definitive or probable bacterial aetiology for infection. The category of severe pneumonia presenting with chest indrawing showed high specificity for lower respiratory infection, and could be used when comparing the burden of respiratory infections in resource poor countries.

## Competing interests

SL, THK, BQ, EAL, PR, ML have no conflict of interest. TP has worked since October 2007 as a part-time medical advisor for GlaxoSmithKline. HN has received honoraria from GlaxoSmithKline Biologicals for consultancies in the past 3 years. ES has received research grants from Pneumo ADIP, WHO, sanofi pasteur and Wyeth Inc, and has received honoraria from Wyeth Inc and GlaxoSmithKline Biologicals. IR has no conflicts of interest.

## Authors' contributions

TP, BQ, THK and EAL conducted the retrospective review of clinical data. TP, SL, BQ and PR designed and monitored collection of clinical data during the vaccine trial. EAL and THK were the clinical study physicians at Bohol Regional Hospital. MGL was the principal investigator of the vaccine trial. HN coordinated the ARIVAC consortium's scientific and administrative activities, and secured the funding. ES and IR were involved in the design of the clinical review process and analysis of it's results. TP, SL, PR, MGL, HN, ES and IR participated in the design of the vaccine trial, development of the analysis plan, and writing of the study report.

All authors have read and approved the final manuscript.

## Pre-publication history

The pre-publication history for this paper can be accessed here:



## Supplementary Material

Additional file 1 Table 1. Table 2. Click here for file
