# Supplementary material for: Clinical case review: A method to improve identification of true clinical and radiographic pneumonia in children meeting the World Health Organization definition for pneumonia
Source: BMC Infect Dis. 2008 Jul 21;8:95. doi: 10.1186/1471-2334-8-95 (PMC2492864; doi:10.1186/1471-2334-8-95)
Supplement: Additional file 1 — Table 1. Table 2. [file 1471-2334-8-95-S1.doc]

Table 1. Summary of clinical data on Filipino children aged from 6 weeks to 23 months hospitalised for an episode of clinical pneumonia defined by the WHO criteria

|  | **WHO- Pneumonia case management classification (%)** | | | |
| --- | --- | --- | --- | --- |
| **Number of episodes** | **Pneumonia**  **N = 290** | **Severe pneumonia**  **N= 785** | **Very severe pneumonia**  **N= 120** | **Total**  **N= 1195** |
| **Sex**   - Male - Female | 168 (57.9)  122 (42.1) | 489 (62.3)  296 (37.7) | 72 (60)  48 (40) | 729 (61.0)  466 (39.0) |
| **Age:**   - 1 to 5 months - 6 to 11 months - 12 – 23 months | 48 (16.6)  103 (35.5)  139 (47.9) | 256 (32.6)  276 (35.2)  253 (32.2) | 41 (34.2)  43 (35.8)  36 (30.0) | 345 (28.9)  422 (35.3)  428 (35.8) |
| **Rectal temperature:**   - <38.0°C - 38.0°C – 39.4°C - ≥ 39.5°C | 70 (24.1)  160 (55.2)  60 (20.7) | 420 (53.5)  306 (39.0)  59 (7.5)) | 58 (48.3)  48 (40.0)  14 (11.7) | 548 (45.9)  514 (43.0)  133 (11.1) |
| **Oxygen saturation:**   - 0_2_ saturation ≥ 90% - 0_2_ saturation < 90% - Not evaluated | 269 (92.8)  17 (5.9)  4 (1.3) | 629 (80.1)  126 (16.1)  30 (3.8) | 67 (55.8)  43 (35.8)  10 (8.4) | 965 (80.8)  186 (15.5)  44 (3.7) |
| **Expiratory wheeze in auscultation**   - Yes - No | 27 (9.3)  263 (90.7) | 400 (51.0)  385 (49.0) | 49 (40.8)  71 (59.2) | 476 (39.8)  719 (60.2) |
| **White blood cell count**   - < 15.0 x10^9^ - ≥ 15.0 x10^9^ - Not evaluated | 191 (65.9)  82 (28.3)  17 (5.8) | 467 (59.5)  230 (29.3)  88 (11.2) | 69 (57.5)  44 (36.7)  7 (5.8) | 727 (60.8)  356 (29.8)  112 (9.4) |
| **C-reactive protein concentration**   - <40 mg/mL - 40- 119mg/mL - ≥ 120mg/mL - Not evaluated | 184 (63.4)  43 (14.8)  26 (9.0)  37 (12.8) | 524 (66.8)  116 (14.8)  25 (3.2)  120 (15.2) | 78 (65.0)  20 (16.7)  6 (5.0)  16 (13.3) | 786 (65.8)  179 (15.0)  57 (4.8)  173 (14.4) |
| **Blood culture (Number)**** | *Salmonella* Typhi *(2), Staphylococcus aureus (2), Streptoccoccus pneumoniae (1)* | *Staphylococcus aureus (1), Streptococcus pneumoniae (2)* | *Neisseria meningitidis (2), Salmonella* Typhi *(1), Staphylococcus aureus (1), Streptococcus pyogenes (1)* | In total 13 |
| **Chest radiograph*****   - Primary endpoint - Other infiltrate - Normal - Not evaluated | 25 (8.6)  40 (13.8)  213 (73.5)  12( 4.1) | 132 (16.8)  139 (17.7)  466 (59.4)  48 (6.1) | 25 (20.9)  27 (22.5)  64 (53.3)  4 (3.3) | 182 (15.2)  206 (17.2)  743 (62.2)  64 (5.4) |
| **Died in hospital or within 14 days from the discharge**   - Yes - No | 4 (0.3)  286 (99.7) | 4 (0.5)  781 (99.5) | 11 (9.2)  109 (90.8) | 19 (1.6)  1176 (98.4) |

*Pneumonia: Cough and/or difficult breathing and respiratory rate >60/min if age <2 months, >50/min if age 2 to 11 months, > 40/min if age > 11 months. Severe pneumonia: Cough and/or difficult breathing and lower chest-wall indrawing. Very severe pneumonia: Cough and/or difficult breathing and inability to drink or central cyanosis.

** Excluding probable non-pathogenic bacteria

*** Primary endpoint consolidation: Presence of a dense opacity that may be a fluffy consolidation of a portion or whole of a lobe or of the entire lung, often containing air bronchograms and sometimes associated with pleural effusion, or a pleural effusion in the lateral pleural space associated with a pulmonary infiltrate or an effusion large enough to obscure such an opacity

Table 2. Results of a retrospective clinical review of episodes of suspected community acquired pneumonia among Filipino children aged from 6 weeks to 23 months

|  | **WHO- Pneumonia case management classification (%)** | | | |
| --- | --- | --- | --- | --- |
| **Clinical review diagnosis** | **Pneumonia**  **N= 290** | **Severe pneumonia**  **N= 785** | **Very severe pneumonia**  **N= 120** | **Total**  **N=1195** |
| Culture confirmed bacterial pneumonia* | 1 (0.3) | 3 (0.4) | 2 (1.7) | 6 (0.5) |
| Suspected bacterial pneumonia* | 24 (8.3) | 87 (11.1) | 17 (14.2) | 128 (10.7) |
| Unspecified pneumonia* | 11 (3.8) | 87 (11.1) | 19 (15.8) | 117 (9.8) |
| Suspected viral pneumonia* | 20 (6.9) | 118 (15.0) | 13 (10.9) | 151 (12.6) |
| Clinical lower respiratory tract infection | 64 (22.1) | 275 (35.0) | 34 (28.3) | 373 (31.2) |
| Bronchiolitis** | 22 (7.6) | 177 (22.5) | 18 (15.0) | 217 (18.2) |
| Upper respiratory infection | 48 (16.6) | 2 (0.3) | 1 (0.8) | 51 (4.3) |
| Gastroenteritis | 37 (12.7) | 12 (1.5) | 3 (2.5) | 52 (4.4) |
| Other diagnosis | 63 (21.7) | 24 (3.1) | 13 (10.8) | 100 (8.3) |

* Clinical review diagnosis of pneumonia was used only if the chest radiograph showed infiltrates in the standard WHO pneumonia interpretation or in the interpretation done by the independent paediatric radiologist (In total 402 episodes)

** Diagnosis of bronchiolitis included also respiratory infections with transient expiratory wheezing not diagnosed as asthma
